# The future of mobility-as-a-service: trust transfer across automated mobilities, from road to sidewalk

**DOI:** 10.3389/fpsyg.2023.1129583

**Published:** 2023-05-12

**Authors:** Jacob G. Hunter, Elise Ulwelling, Matthew Konishi, Noah Michelini, Akhil Modali, Anne Mendoza, Jessie Snyder, Shashank Mehrotra, Zhaobo Zheng, Anil R. Kumar, Kumar Akash, Teruhisa Misu, Neera Jain, Tahira Reid

**Affiliations:** ^1^School of Mechanical Engineering, Purdue University, West Lafayette, IN, United States; ^2^Industrial and Systems Engineering, San Jose State University, San Jose, CA, United States; ^3^Honda Research Institute USA Inc., San Jose, CA, United States

**Keywords:** automated vehicles (AVs), trust in automation, dual mobility, semi-structured interview, mobility-as-a-service (MaaS)

## Abstract

While trust in different types of automated vehicles has been a major focus for researchers and vehicle manufacturers, few studies have explored how people trust automated vehicles that are not cars, nor how their trust may transfer across different mobilities enabled with automation. To address this objective, a dual mobility study was designed to measure how trust in an automated vehicle with a familiar form factor—a car—compares to, and influences, trust in a novel automated vehicle—termed sidewalk mobility. A mixed-method approach involving both surveys and a semi-structured interview was used to characterize trust in these automated mobilities. Results found that the type of mobility had little to no effect on the different dimensions of trust that were studied, suggesting that trust can grow and evolve across different mobilities when the user is unfamiliar with a novel automated driving-enabled (AD-enabled) mobility. These results have important implications for the design of novel mobilities.

## 1. Introduction

Urban transportation services are envisioning a shared vehicle future centered around Mobility as a Service (MaaS) that meets transport needs through a single interface offered by a service provider. It combines different transportation modes for the user in a tailored mobility package, like a monthly mobile phone contract (Hietanen, 2014). It may involve shared mobilities, defined as “the shared use of a vehicle, bicycle, or other low-speed modes that enables users to have short-term access to transportation modes on an ‘as-needed' basis” (Shaheen et al., 2015). At the same time, increases in vehicle automation, such as advanced driver-assistance systems (ADAS) and automated driving systems (ADS), are introducing more complexity into the personal transportation landscape. In fact, a convergence between automated vehicle (AV) technology and shared mobility is beginning to develop with small-scale shared automated vehicle tests around the world (Stocker and Shaheen, 2017).

### 1.1. Shared automated vehicles

Shared mobility is evolving as cities around the world repurpose traditionally “car-centric” public spaces to accommodate a variety of road users. Several challenges emerge for policymakers and researchers alike in developing a shared mobility ecosystem, including the need for multimodal integration and accessibility for all. With the advancement of automated vehicle (AV) technology, shared mobility services may provide important alternatives to conventional transportation and have the potential to alter the way in which people move in and around cities. While shared automated mobility has a role to play in the future of transportation, much research on its usage, impact on travel behavior, traffic congestion, and environmental impact requires exploration (Stocker and Shaheen, 2017). Another challenge that remains with respect to shared automated vehicles (SAVs) is developing a coherent understanding of how users will trust such systems (Mittendorf, 2017; Hartl et al., 2018) so that strategies for effective calibration of user trust can be developed. Moreover, most SAVs will be used on an “as-needed” basis, which may have another impact on the trust and adoption of the mobility. It is therefore incumbent upon researchers to study users' trust in different types of automated mobilities as well as how this trust may change when switching from one automated mobility type to another. In the next section, constructs of trust in automation are discussed.

### 1.2. Trust in automation: theoretical models and measurements

Trust in automation has been studied for decades, and yet it remains a challenging topic for the wider research community. Persistent questions of how trust can be defined, established, and measured are areas of interest among researchers. One of the most accepted theories of how trust is established is given by the dyadic model of trust proposed by Mayer et al. (1995). The model defines trust as “the willingness to be vulnerable to the actions of others based on the expectation that the other will perform a particular action important to the trustor irrespective of the ability to monitor or control”. According to this model, trust depends on individual propensity (or general willingness) to trust others and the trustworthiness of the party to be trusted (trustee). A person's trust propensity results from different developmental experiences, personality types, and cultural backgrounds and determines how much a person trusts a trustee prior to their having any knowledge of that individual (Hoff and Bashir, 2015). The model establishes that trust is based on an individuals' ability to trust, benevolence to the extent to which a trustee is perceived to want to do good, and the integrity with which the trustee consistently adheres to a set of principles that the trustor finds acceptable. The ability to take risks is then the behavioral manifestation of the willingness to be vulnerable, i.e., the outcome of trust.

While this model establishes a strong working definition of the theoretical construct of trust, Lee and See (2004) helped further strengthen the understanding of how trust is established when humans interact with automation technology. They distilled the model of trust proposed by Mayer et al. (1995) into three dimensions: performance, process, and purpose. Performance seeks to define the current and previous operation of the automated system and how it comprises characteristics such as reliability, competency, and ability. Process describes how the automation matches the operators' objectives, which matches the integrity aspect. Finally, purpose describes the developers' intention to possess a positive orientation toward the operator, which aligns with the benevolence in Mayer et al. (1995). The comparison of these models has been described in great detail by Körber (2019) and forms the basis of our understanding of trust in automation.

Körber (2019) argues that trust is characterized by a static individual component, wherein humans rely on their inherent propensity to trust, depending on individual differences such as developmental experiences, personality type, and cultural backgrounds. This theoretical approach seeks to combine the constructs of interpersonal trust as well as trust in automation, which considers individual differences. The premise of this approach is to consider how *reliable performance, predictable outcomes*, and *positive intentions of developers* are factors of perceived trustworthiness, while prior experiences with automation (familiarity), individuals' *propensity to trust* in automation, and their general *trust in automation* act as moderating factors that influence perceived trust. Thus, trust in automation is defined as: “the attitude of a user to be willing to be vulnerable to the actions of an automated system based on the expectation that it will perform an action important to the user, irrespective of the ability to monitor or to intervene.” This definition implies that trust is more than the act of trusting performance, but rather a general attitude that the human is willing to be vulnerable based on their inherent state of trust (Mayer et al., 1995). In this paper, we adapt a survey developed by Körber (2019) and analyze each participants' trust along the dimensions italicized above. In the next section, individual preference in automated driving style is discussed.

### 1.3. Individual preference in automated driving style

While most autonomous vehicles (AVs) exhibit a default driving style, the default may not provide the most comfortable user experience for every individual. The mismatch between the AV's driving style and the user's expectation could result in lower trust in automation, thereby impeding the successful adoption of different SAVs (Trende et al., 2019). One approach toward fulfilling the expectation of the user is by tailoring the AV to match the style in which they would like to be driven. Indeed, several studies have demonstrated how a specific driving style can help improve the user's perception of an AV and contribute toward building trust (Ekman et al., 2019). It has been shown that fulfilling the drivers' maneuver preferences can lead to better comfort for them in an automated vehicle (Bellem et al., 2018). In fact, this level of personalization is not a new concept and has been approached by adopting preferences, patterns, styles, and skills of drivers in AVs (Hasenjager and Wersing, 2017; Natarajan et al., 2022).

Based on this evidence, we explicitly consider driving style preference in the proposed dual mobility experiment. Furthermore, we ensure that the driving style is consistent throughout nominal (standard) driving events and during “conflict events” in which the automated mobility demonstrates advanced decision-making while ensuring the safety and reliability of the vehicle. In the next section, we present the complete experiment design along with the study procedure and analysis methods used.

### 1.4. Aims of the study

While promising, a critical challenge facing the adoption of a MaaS ecosystem is that many users may have infrequent or intermittent interactions with a variety of different shared vehicles and mobilities. It is well-accepted that user trust is critical for successful adoption of automated technologies (Lee and See, 2004; Parasuraman et al., 2008; Chen et al., 2010; Hancock et al., 2011; Cho et al., 2015). Therefore, it is necessary to understand users' trust in automation while experiencing new modes of automated mobility. More specifically, for a multimodal transportation ecosystem, it is critical to understand how trust in one automated mobility affects the trust in *another* automated mobility. Existing trust transfer theory in e-commerce suggests that trust can be transferred from one trusted source to another unknown target if there is a specific association between them (Doney and Cannon, 1997; Stewart, 2003). However, such a theory has not been studied in the context of interactions with automated vehicles. In particular, given the dynamic nature of trust (Cho et al., 2015) during a continuous interaction with automated vehicles, it is possible that the existing trust in one mobility not only influences the initial trust in another mobility but also influences how trust evolves in the latter mobility.

This raises the following research questions.

1. How does human trust evolve in one automated mobility vs. another automated mobility with no prior interaction with either of the mobilities?
2. Does prior experience in an automated car influence trust in a novel automated mobility (that is not a car)?

To address these questions, a novel dual mobility simulator experiment was designed that elicited participants' trust responses in two different automated driving (AD) systems. The two mobilities were a car and a sidewalk mobility. The car enabled with AD was a regular passenger car, whereas the sidewalk mobility was a personal-automated mobility typically used in urban environments which share space with pedestrians. Throughout the experiment, responses to trust questionnaires helped gauge how participants' trust changed with varying drives. Participants then shared their thoughts in a semi-structured interview at the conclusion of the experiment.

Our prior work showed (through a limited quantitative analysis of a subset of participants) whose trust transfers across mobility types based only on baseline and tutorial measurements (Mehrotra et al., 2023). In the previous paper, findings confirmed that aggressive style participants had a higher reliability when moving from car to sidewalk mobility. Additionally, it was found that the tutorial helped increase trust of aggressive participants in sidewalk mobility. In this paper, we expound upon the prior work by considering additional research questions regarding trust in automation, considering quantitative data from all experiment participants, and providing a rigorous thematic analysis of the qualitative data collected via semi-structured interviews with each participant.

The goal of this research is to evaluate how trust evolves in one automated mobility vs. another automated mobility with no prior interaction with either of the mobilities, and whether prior experience in an automated car influences trust in a novel automated mobility (that is not a car).

## 2. Materials and methods

### 2.1. Experiment overview

All participants completed two experimental sessions (one on each mobility) in a single day. For logistical reasons related to changing the mobility platform from car to sidewalk mobility and vice versa, a 3-h gap between the sessions was observed. The driving style of the mobility (defensive or aggressive) was chosen based on the participants' responses in the screening survey, as described in Section 2.5.1. This ensured that participants had the best chance to increase their trust in the mobilities.

In each experimental session, participants experienced three simulated drives: a tutorial drive, a standard drive, and a proactive drive. The three drives are explained in detail below, using terminology from Table 1.

1. The tutorial drives consisted of navigation through an empty map, i.e., the absence of traffic participants (e.g., cars or pedestrians). The purpose of this drive was to familiarize the participant with the respective mobility.
2. The “standard drives” consisted of an AD vehicle navigating standard events, or events where the AD decision-making is unambiguous (e.g., driving through a 4-way stop intersection with a clear order of who has the right-of-way). Additionally, the AD vehicle drove in a manner consistent with the user's preference (defensive or aggressive). These intentional design decisions were to demonstrate reliable mobility behavior such that users would develop the reliability aspect of trust, as measured and described prevalently in the literature (Beggiato and Krems, 2013; Beggiato et al., 2015; Häuslschmid et al., 2017; Akash et al., 2020a; Azevedo-Sa et al., 2021; Hunter et al., 2022).
3. The “proactive drives” consisted of an AD vehicle engaging in proactive behavior in order to navigate conflict events, or events where the AD decision-making is ambiguous (e.g., overtaking or not overtaking a vehicle ahead). Proactive behavior is a function of actions, either defensive or aggressive, based upon the driving style preference of the participant. The conflict events were designed in order to elicit a trust response as the vehicle exhibited proactive behavior while maintaining an environment where the automation is both safe and reliable. Therefore, the “proactive drives” were a sequential step in building trust beyond the reliability trust stage of the “standard drives,” similar to building trust from the dependability stage to the faith-based stage in interpersonal trust (Rempel et al., 1985). Table 2 contains the eight conflict events which participants encountered during the “proactive drives.” Since natural differences existed between the car and sidewalk mobility simulations, equivalent conflict events were created to match each other as close as possible.

**Table 1:** Terminology used to describe the study design and procedure.

**Key word**	**Definition**
Event	Situation where the AD vehicle must interact with other traffic participants (TPs) including other cars and pedestrians.
Conflict event	*Event* where the AD vehicle must make a decision (e.g., by waiting, yielding, or overtaking) in order to continue smooth driving. In such events, the traffic rules allow more than one legal maneuver (e.g., overtake or not to overtake).
Standard event	*Event* that is not a conflict event (e.g., driving through a 4-way stop intersection with clear order of right-of-way).
Action	How AD vehicle reacts to an *event*. For a *conflict event*, the AD vehicle can react aggressively or defensively. All actions are assumed to be legal and/or common behaviors.
Aggressive action	AD system *action* that prioritizes driver more than interacting TPs. Aggressive action typically leads to shorter travel time (e.g., over-taking, insisting right of the way etc.). Choosing this action often leads to narrower margins with other TPs.
Defensive action	AD system *action* that prioritizes TPs more than ego-vehicle. This action typically leads to increased margin between ego-car and interacting TPs (e.g., yielding, giving right of way to others etc.). Choosing this action often leads to increased travel time.
Proactive behavior	The way the AD vehicle resolves *conflict events* based on *actions* that are matched to a user's preferred driving style (aggressive or defensive). Decisions are “proactively” made without user's permission, but the user is informed about AD vehicle's *action* using auditory and visual cues to enhance automation transparency.
Drive	A single sequence of multiple *events* consisting of *conflict events* and/or *standard events*. Each *drive* is approximately 10 min long and may involve 8 *conflict events*.
Session	A group of *drives* on the same mobility, linked together by brief pauses/transitions. There were two sessions for each participant: session 1 (morning) and session 2 (afternoon).

**Table 2:** List of conflict events.

**Car events**	**Sidewalk mobility events**
Stale green light	Stale green light
Simultaneous arrival at intersection	Simultaneous arrival at intersection
Right turn merge (red light)	Merge into crowd of pedestrians
Left turn yield (green light)	Yield to turning car
Car sudden backout from driveway	Pedestrian runout from behind house
Jay walking pedestrians	Jay walking pedestrians
Passing slowing car	Passing slow pedestrians
Cars blocking path	Pedestrians blocking path

**Figure 1:**
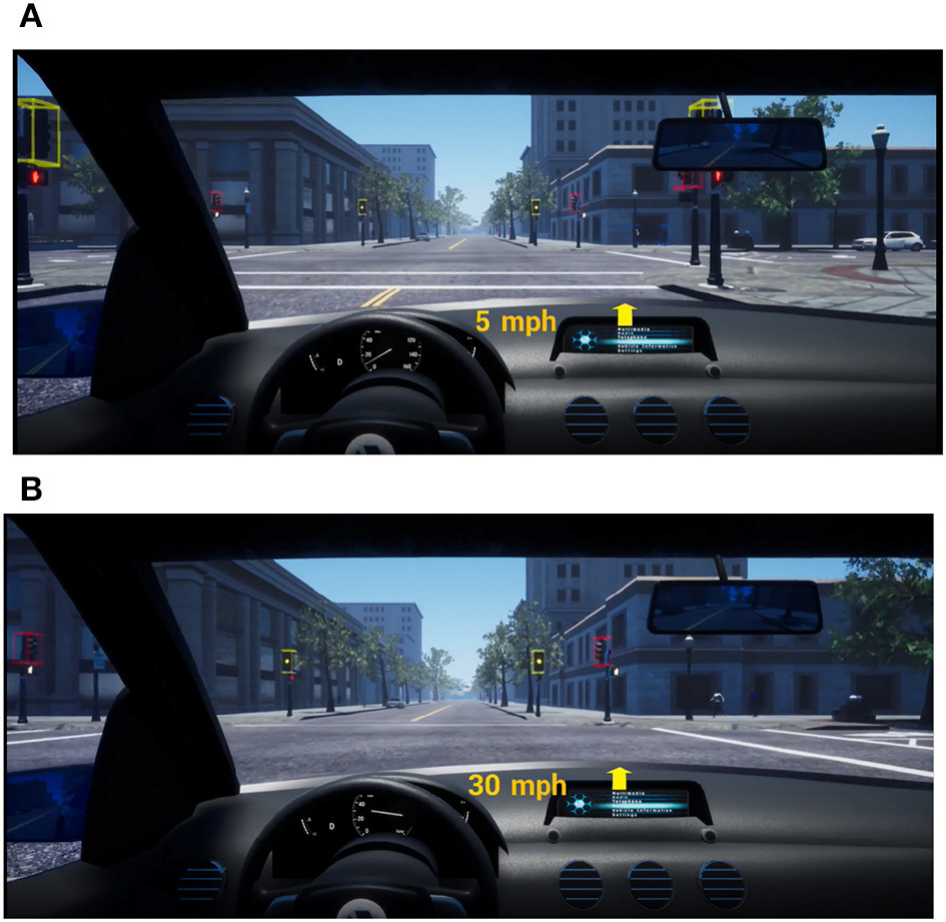
Proactive behavior of AD at the stale green (green then yellow) event. **(A)** Defensive: slowing once the light turns yellow. **(B)** Aggressive: maintaining speed once the light turns yellow.

A consistent approach to automation transparency was employed throughout the experiment to foster situational awareness and draw attention to proactive behavior. Two forms of transparency were used. First, augmented reality (AR) bounding boxes were used to highlight pedestrians, traffic signals, and other vehicles. When a traffic signal was highlighted, the color of the bounding box matched the color of the traffic signal. Second, at each conflict event, the system played a chime sound, followed by an audio (text-to-speech-based) notification. For example, for the “stale green light” conflict event shown in Figure 1, the speech notification for the aggressive proactive behavior said, “yellow light ahead; continuing” while that for the defensive proactive behavior said, “yellow light ahead; stopping.” To emphasize the color change of the signal, the AR bounding box highlighting the traffic light changed color from green to yellow.

### 2.2. Participants

Forty-eight participants were recruited for the study from and near San Jose, California. They were recruited through Craigslist jobs, social media, and a university research experience pool. Each applicant completed a screening survey in order to participate in the research. All participants were required to (1) be legally allowed to drive in the United States; (2) be 18 years of age or older; (3) have no self-reported hearing impairment; (4) have normal or corrected-to-normal vision using contact lenses (glasses could not be accommodated due to the virtual reality headset); and (5) not be easily susceptible to motion sickness. Additionally, a COVID-19 questionnaire was included to prevent anyone infected from participating. All 48 participants who completed the study were compensated $125; participants who failed to complete the study due to motion sickness or other constraints were compensated $25. The study was approved by the Institutional Review Board at San Jose State University.

### 2.3. Equipment

**Figure 2:**
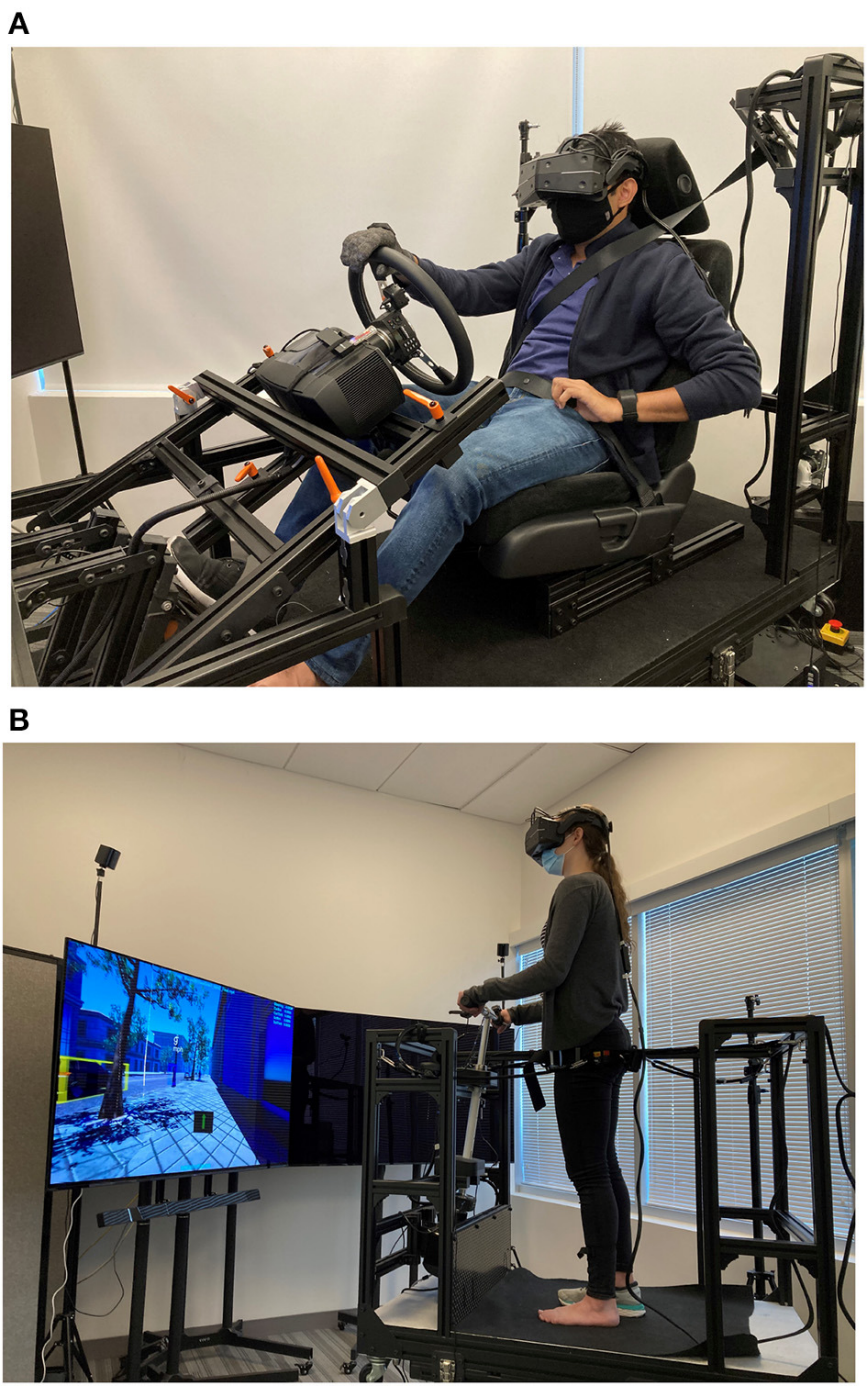
Platforms for the car and sidewalk mobility mounted on the motion base. **(A)** Car platform. **(B)** Sidewalk mobility platform.

A wide field-of-view (FoV) is necessary for a driver to observe events at an intersection; therefore, a VR headset with a broad FoV was chosen (Goedicke et al., 2018). Specifically, a StarVR headset with a 210-degree FoV was used in the study. A motion base (MB-200 6-degree of freedom motion base by Cosmate Co., Ltd.) was used to create a high-fidelity simulation in which participants could feel the typical forces experienced in a vehicle. The car and sidewalk mobility platforms were mounted upon the motion base, as seen in Figure 2. The automated driving was simulated by replaying a past researcher's drive via the “Wizard of Oz” technique (Wang et al., 2017). The display and audio were rendered using Unreal Engine 4.24 (Epic Games, 2019) with AirSim (Shah et al., 2018) that consisted of a custom city environment including traffic lights, other vehicles, pedestrians, stop signs, and roundabouts. Each drive was pre-recorded as a stereoscopic 360-degree video with a resolution of 4096 × 4096 along with the vehicle's position, orientation, and steering input. The recorded drives were played on the StarVR headset using a custom software developed in Unity (Unity Technologies, 2019) that synchronously controlled the motion base and the steering wheel to mimic the vehicle's behavior in the recording.

### 2.4. Design of the experiment

**Figure 3:**
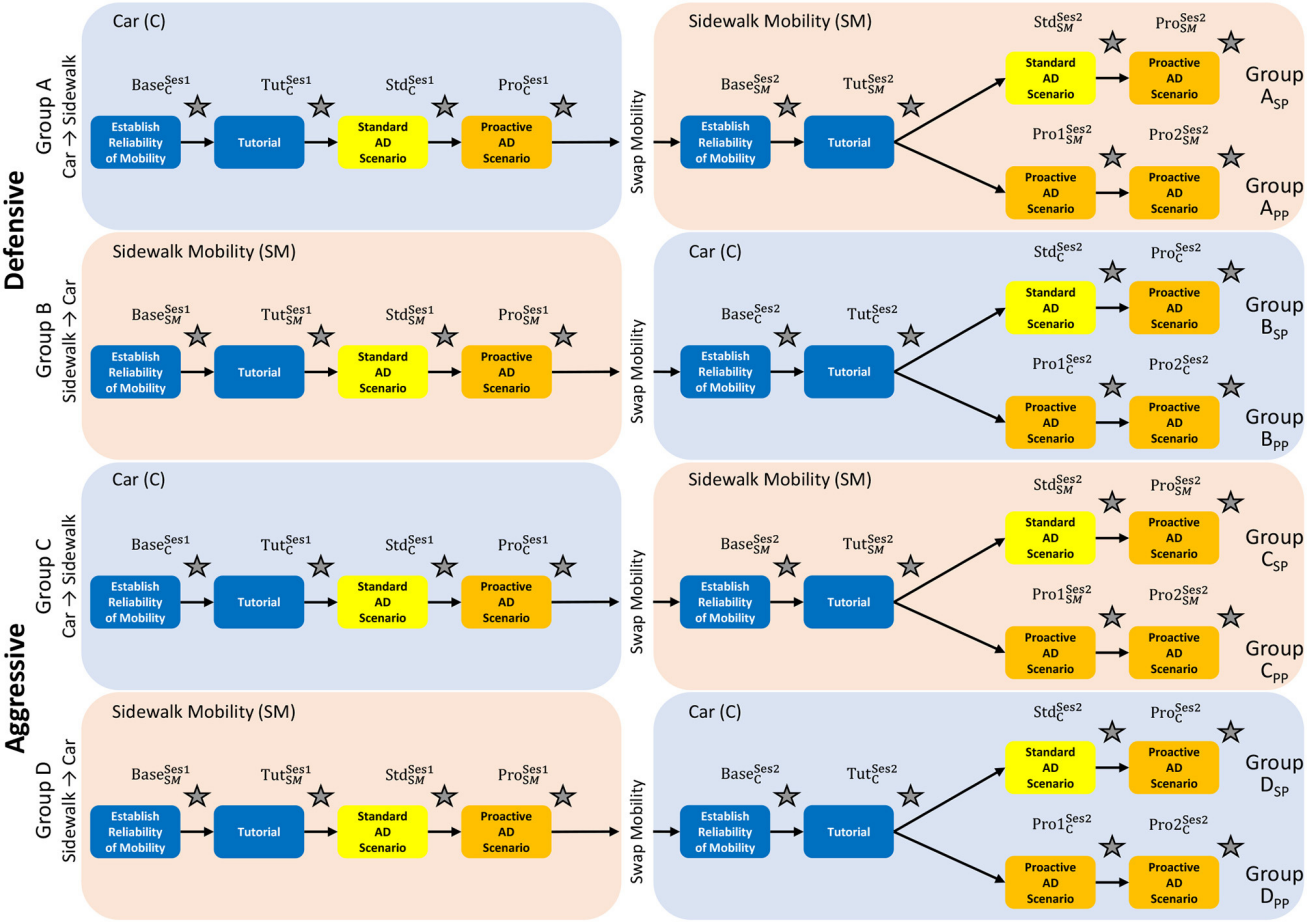
Study design.

For this experiment, there were 3 between subject factors: (1) Driving style (Aggressive or Defensive) (2) Order of mobility (Car to sidewalk or Sidewalk to Car), and (3) Order of treatment in the afternoon session (Tutorial → Standard → Proactive or Tutorial → Proactive → Proactive). A 2 × 2 × 2 between-subject experiment was considered for the experiment design. Twenty-four participants experienced the aggressive driving style, whereas the other 24 participants experienced the defensive driving style. For each driving style, 12 participants experienced the car (morning session) first, followed by the sidewalk mobility (afternoon session). The remaining 12 participants experienced the sidewalk (morning session) first, followed by the car mobility (afternoon session). In the afternoon sessions, for all driving styles and mobilities, 6 participants experienced the “standard” drive followed by the “proactive” drive. The other 6 participants experienced “proactive” drives twice. The complete study design is summarized in Figure 3. We considered ways to control for several confounding variables for the experiment: (1) lack of familiarity with the simulator environment was mitigated by providing tutorial drives to get participants accustomed to the environment; (2) to ensure attention, the participants were encouraged to speak throughout the experiment describing how they felt, which kept them engaged with the study; and (3) to ensure participants were familiar with traffic conventions, only participants with valid driver's license was included.

#### 2.4.1. Power analysis

While several factors were evaluated, it was important to ascertain the number of participants that would be needed for the experiment. For this purpose, a power analysis was done. While aiming for a large effect size (d = 0.45), the between-subject design (style preference) was chosen. The type of session (morning/afternoon), and the drive type (tutorial, standard event, conflict event) were the within-subject factors. Owing to this, a power of 80% was ensured by selecting 24 participants for each driving style. While this was sufficient for comparisons around assessing the effects of the sessions and mobilities, comparing drives across sessions for standard events and conflict events in the session left with only considering comparisons across 6 participants, which is underpowered. The power analysis was done using the “Pangea” power analysis tool (Westfall, 2015).

### 2.5. Procedure

The study procedure consisted of four parts: a screening survey, a morning session with one automated mobility followed by a gap, an afternoon session with a second automated mobility, and finally a semi-structured interview. Additional details are provided in the following subsections.

#### 2.5.1. Screening phase

The screening survey included a short video with questions to determine the preferred driving style (defensive or aggressive) of the participant. The video consisted of two short drives with different driving styles, which were recorded from the dashboard of a real vehicle. For the defensive driving style, the video showed the car driving at the stated 25 mph speed limit, following at least 3 car lengths behind the preceding vehicle, and accelerating smoothly. For the aggressive driving style, the video showed the car driving slightly above the stated 25 mph, following a preceding vehicle by less than one car length, and accelerating abruptly. Participants were then asked to indicate the style in which they preferred to be driven. Note that this style could differ from their *own* driving style. This procedure was introduced to avoid confounding effects of perceived capability and driving style mismatch on participant trust. Based on the screening, 48 participants were assigned to one of the two driving styles of their choosing. Apart from the driving style, participants provided their informed consent. They also completed an Advanced Driver-Assistance Systems (ADAS) survey.

#### 2.5.2. Driving sessions: morning and afternoon

**Figure 4:**
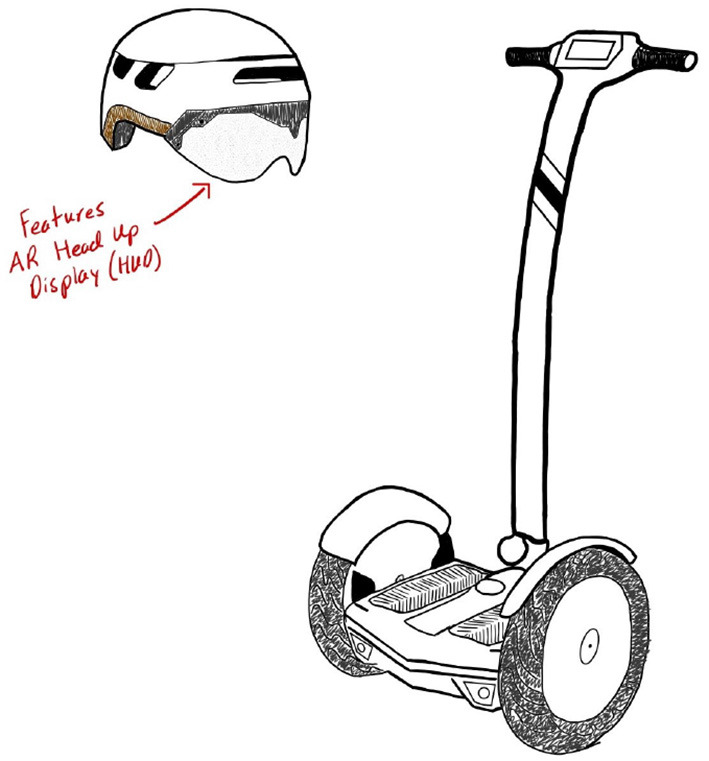
Sketch of the sidewalk mobility concept shown to participants before their drives.

Participants experienced two sessions with automated vehicle simulators, with a 3-h gap between sessions. If participants experienced sidewalk mobility in the morning, they experienced the automated car in the afternoon and vice versa. Before a participant used the sidewalk mobility, they were also shown the sketch in Figure 4 in order to provide them with a reference of what kind of vehicle they were riding. The morning and afternoon sessions generally followed the same structure, with some notable differences. At the beginning of each session, participants were given a short briefing regarding the respective mobility. The experimenter gave an overview of the mobility's controls and informed participants that the hardware and software were 100% reliable; however, participants were instructed to monitor the automated driving since the mobilities in this experiment are considered SAE Level 2 vehicles (SAE International, 2021). Despite the vehicles being 100% reliable, not all participants *perceived* them as 100% reliable due to individual differences in driving experience and preference. Therefore, as part of the briefing, participants were informed that their “intent to takeover” would be recorded by the mobility but the mobility would not respond (due to our simulator limitations). After the briefing in the morning session, the participants experienced three drives, namely, “tutorial,” “standard,” and “proactive” (described in Section 2.1). In the afternoon session, they were again given a briefing followed by three drives; however, after the tutorial, half of the participants received a “standard” then “proactive” drive on the second mobility while the other half received two consecutive “proactive” drives. The swapping of different mobility types allowed for the observation of trust transfer from one type to the other (i.e., car to sidewalk mobility; sidewalk mobility to car). Additionally, dividing participants to compare “standard → proactive” vs. “proactive → proactive” was done to observe if changing the drive type within a given mobility after a mobility change impacted trust in automation.

Trust was measured four times per session as indicated by the gray stars in Figure 3. Each gray star is labeled based on the drive, mobility, and whether the drive was before or after the mobility swap. For example, ${\text{Tut}}_{\text{C}}^{\text{Ses}2}$ refers to survey responses collected after the tutorial drive for the car mobility and in session 2 (i.e., swapped from sidewalk to car). In order to capture different aspects of trust pertinent to the research interests of this study, we adapted a trust in automation questionnaire developed by Körber (2019). Although Körber (2019)'s trust questionnaire originally contained six dimensions of trust, we only used five dimensions and excluded “familiarity”. In their theoretical model, familiarity itself is not considered to be an element of trust in automation but indirectly influences it as a moderator. As recommended by Körber (2019), we eliminated the “familiarity” focus on the “core questionnaire”. The adapted trust survey questions and their respective dimensions are shown in Table 3.

**Table 3:** Trust in automation survey (adapted from Körber, 2019).

**Item**	**Scale**
The vehicle is capable of interpreting situations correctly	Reliability/competence
The vehicle works reliably	Reliability/competence
A vehicle malfunction is likely	Reliability/competence
The vehicle is capable of taking over complicated tasks	Reliability/competence
The vehicle might make sporadic errors	Reliability/competence
I am confident in the vehicle's self-driving capabilities	Reliability/competence
The vehicle's state is always clear to me	Understanding/predictability
The vehicle acts unpredictably	Understanding/predictability
I am able to understand why the vehicle responds in certain ways	Understanding/predictability
It's difficult to identify what the vehicle will do next	Understanding/predictability
The vehicle's developers are trustworthy	Intention of developers
The vehicle's developers take my wellbeing seriously	Intention of developers
I should be careful with the self-driving vehicle	Propensity to trust
I'm prone to trusting the self-driving vehicle than mistrusting it	Propensity to trust
The self-driving vehicle generally works well	Propensity to trust
I trust the vehicle	Trust in automation
I can rely on the vehicle	Trust in automation

#### 2.5.3. Post-completion interview

A semi-structured interview was conducted at the conclusion of the study with each participant; the purpose was to understand how trust transferred between mobilities, how initial trust may have evolved, perceived differences between the car and sidewalk mobility, and opinions on the voice assistance during conflict events. The interview involved pre-defined questions for all participants but allowed the researchers to ask follow-up questions on an individual basis. Participants were interviewed about certain behaviors they exhibited during the study sessions in order to gain additional insight into their thoughts and feelings about the simulation. This format allowed supplemental findings to be obtained in addition to investigating the main research questions. Following the interview, participants were compensated for their time. The interviews were recorded so that an audio record was available for later analysis.

### 2.6. Data analysis

#### 2.6.1. Quantitative methods

A linear mixed effects model is used to understand the development of trust over participants' first interaction with the mobility. The model has two independent factors, an interaction factor, and one random factor. The independent factors are drive type and mobility mode. The drive type is the instance of the repeated measure of trust survey taken for each drive (including after the “baseline,” or “briefing”). Specifically, the drive type can be baseline, tutorial, standard, or proactive (refer to the gray star location in Figure 3). The interaction factor is simply the interaction between drive type and mobility mode. Lastly, the random factor is the participant. The model allows comparisons to be made between participants on the car vs. those on the sidewalk mobility, as well as accounts for the repeated measurements of trust for each drive type.

An omnibus ANOVA is performed to identify the significant factors influencing trust, with subsequent Tukey pairwise comparisons to understand the exact relations between levels. Shapiro-Wilk test (Shapiro and Wilk, 1965) was performed to determine if the sample did not violate the normality assumption. Findings are reported for those who preferred and experienced the defensive style automated driving (AD) as well as the aggressive style AD using separate models for each driving style.

#### 2.6.2. Qualitative methods

All interviews were recorded, and the audio files were transcribed using otter.ai software (Otter.ai, 2022). Two researchers reviewed the transcribed text for the accuracy and validity of the software-generated transcripts. The resultant text data was used to conduct a thematic analysis (Braun and Clarke, 2006). The thematic analysis aimed at addressing three research themes: level of initial trust, preference between mobilities, and trust transfer. As such, the researchers developed the coding scheme to assist in the analysis as shown in Table 4. A coding scheme was created for thematic analysis in this study to investigate the research themes (initial trust level, mobility preference, and trust transfer). The coding scheme was used by three researchers who assigned relevant text from the transcripts to the appropriate research themes independently. The coding was also validated by other researchers to ensure reliability and consistency in the rating. If any discrepancies arose, the researchers discussed and resolved conflicts to arrive at a consensus. After initial calibration, two researchers coded the interview files, and a third researcher verified the codes for accuracy as a quality check. The overall consensus in coding was found to 87.5%. Based on the coding scheme presented in Table 4, some participants were excluded for a given research theme. We report the number of participants (*k*) included in a given level of code in the coding scheme out of the total number of participants (*n*) included in the theme as *k*/*n*.

**Table 4:** Coding scheme used for the thematic analysis.

**Research theme**	**Level(s)**	**Example phrase(s)**	**Inclusion**	**Exclusion**
Level of initial trust	State of trust	“I started off with a pretty high level of trust”	•Explicitly or implicitly stated their level of trust for the beginning of the first drive	•Participants may have stated their level of trust but were too vague as to at what point in the study they experienced it
	State of distrust	“When I started I didn't trust it”	•Keywords for trust: confidence, trust, comfort	
	Neutral	“I think my trust generally was neutral at the beginning.”	•Keywords for distrust: unsure, hesitant, skeptical, doubtful, shaky,	
Preference between the two mobilities	Car	•“I think I trusted the cars capability for the self driving more than the scooter ”	•Explicitly or implicitly stated which they trust or preferred more	•Did not make any type of judgment of which they trusted more
	Sidewalk mobility	“The car was more difficult”		
Trust transfer	Trust transferred across mobilites	“Yeah, it definitely did [transfer].”	•Responded affirmatively or negatively when asked if their trust transferred across mobilities	•Did not respond to the question or were not asked this question
	Trust did not transfer across mobilities	“I actually separated the two.”	•Stated they saw the two mobilites as separate experiences	

## 3. Results

In this section, we answer the proposed research questions based on our analysis of the qualitative and quantitative data. The results include emergent themes from the semi-structured interviews along with key findings from the trust survey responses. Lastly, several participants had thoughts on the automation's transparency, which are synthesized and presented.

### 3.1. Trust evolving in one mobility vs. another with no prior interaction

Thirty-three of forty-eight participants reported whether or not they initially trusted their mobility during session 1 (see Table 5). Six participants, for both the car and sidewalk mobility, reported an initial state of *trust* in their respective mobility. However, ten participants reported an initial state of *distrust* in the sidewalk mobility compared to seven car mobility participants. This section focuses on answering the research question of how trust evolves in each of the mobilities beyond these initial perceptions during session 1.

**Table 5:** Number of participants who reported initial level of trust.

	**Car mobility**			**Sidewalk mobility**		
	**Trust**	**Distrust**	**Neutral**	**Trust**	**Distrust**	**Neutral**
Defensive	3	3	2	0	4	1
Aggressive	3	4	1	6	6	0
Total	6	7	3	6	10	1

#### 3.1.1. Perceived risk associated with the sidewalk mobility in session 1

Fifteen of twenty-four participants who experienced the sidewalk mobility in session 1 shared one or more concerns on four major topics relating to the safety and handling of the sidewalk mobility during their semi-structured interview. First, five participants reported concerns about the sidewalk mobility offering little physical protection from the outside world. For example, Participant 17 said in response to the sidewalk mobility not stopping at a crosswalk “*I think personally...if it just slowed down a little bit more, I feel like I would have trusted it.”* Second, five participants made reference to the sidewalk having unpredictable pedestrians. For example, Participant 20 commented, “*because the sidewalks, like people can come at you more unexpectedly, like through doors and stuff like that.”* Third, eight participants commented on the sidewalk mobility driving dangerously by taking sharp or scary turns, going up and down sidewalks unsafely, or going around blind corners. Participant 45 said about turns with blind spots, “*It was rather quicker than I expected. It was turning at...eight or nine mph. Sometimes. I mean, I don't remember the exact number, but it's quite fast for that.”* Finally, four participants commented on the overall bumpiness of the sidewalk mobility. This is likely due to the design of the sidewalk mobility simulations where the vehicle must ride up and down curbs to cross intersections. Participant 14 noted that the sidewalk mobility was “*shaky, because one, I've never been on this type of vehicle that was autonomous.”* Despite more than half of the participants citing these risks and dissatisfaction with the mobility, several of them shared that their trust in the sidewalk mobility increased throughout subsequent drives during session 1.

#### 3.1.2. Trust evolution associated with the car mobility in session 1

Similarly to those who began on the sidewalk mobility, several participants who used the car mobility in session 1 had misgivings about certain aspects of the automated driving. Participant 5 found the braking to be unreliable such that “*it would...jolt the car back”* while Participant 6 felt the car approached crosswalks with pedestrians too quickly and “*would have started slowing down a bit earlier”*. Nevertheless, twelve of the 24 participants who experienced the car mobility in session 1 (including Participants 5 and 6) expressed in their semi-structured interview that their trust grew as time passed. For example, Participant 1 stated, “*I remember when I was doing the baseline survey for the car one, [and] I was not completely sold on that idea, that it could handle all the complex situations...so like when I went through those situations, like the more time I spent with the system itself, I grew to like [it], trust it more and [have] more confidence in its abilities.”* Participant 33, commented that one of their greatest increases in trust came from seeing the car react to a complex situation before they could react themselves. Similarly, two other participants' trust grew in the mobility from seeing it handle complex situations. Therefore, it appears complex events can be important in trust evolution; however, perhaps more important is repeated experience with the car mobility.

#### 3.1.3. Findings from the survey responses

**Figure 5:**
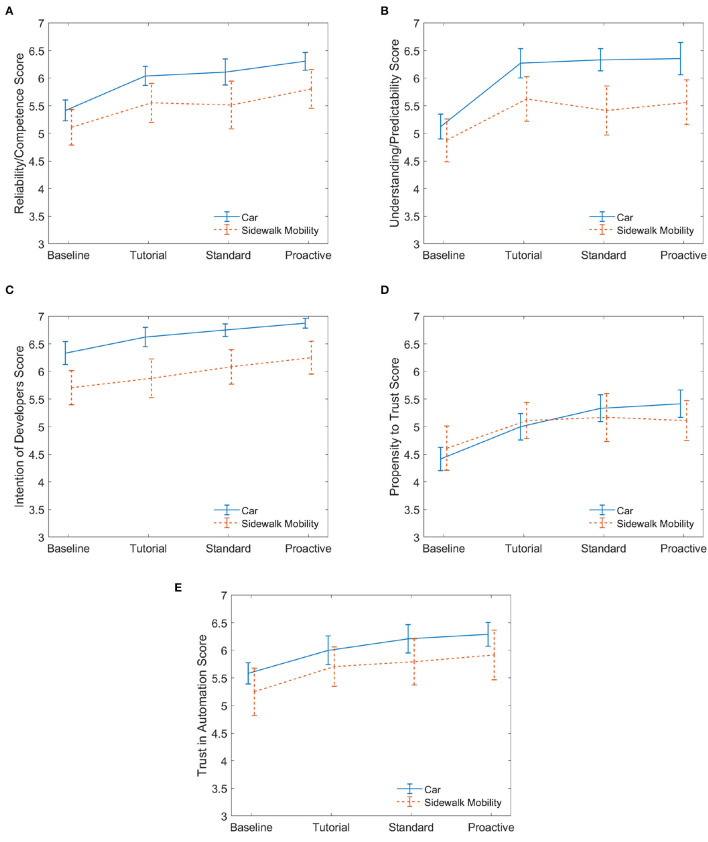
Defensive. These plots for session 1 compare the five dimensions of trust for those who began on the automated car to those who began on the automated sidewalk mobility. Twelve participants experienced the car while another 12 experienced the sidewalk mobility in the first interaction. Mean values are reported with error bars representing the standard error across participants. **(A)** Reliability. **(B)** Predictability. **(C)** Intention. **(D)** Propensity. **(E)** Trust.

**Figure 6:**
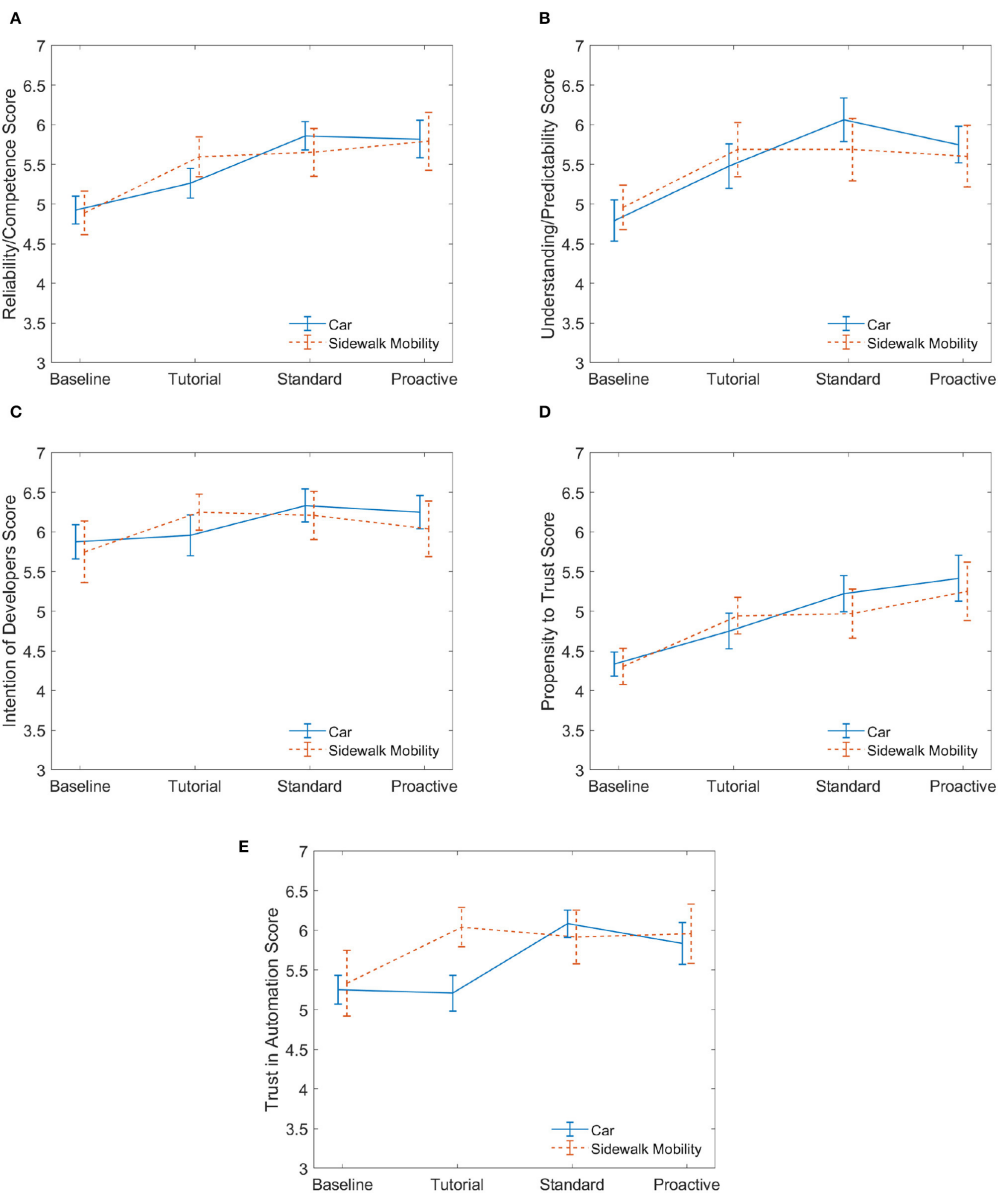
Aggressive. These plots for session 1 compare the five dimensions of trust for those who began on the automated car to those who began on the automated sidewalk mobility. Twelve participants experienced the car while another 12 experienced the sidewalk mobility. Mean values are reported with error bars representing the standard error across participants. **(A)** Reliability. **(B)** Predictability. **(C)** Intention. **(D)** Propensity. **(E)** Trust.

While the semi-structured interview responses yielded interesting insights about the acceptance of the mobilities, the trust survey measurements from session 1 allowed us to compare quantitatively the responses of participants that began with the sidewalk mobility to those who began with the car mobility. For the participants in the defensive group, Group B (sidewalk first) was contrasted with Group A (car first). In like manner, Group D (sidewalk first) was contrasted with Group C (car first) for the aggressive group. As previously mentioned in Section 2.6.1, we used a linear mixed effects model followed by Tukey's test to analyze the effect of the mobility and the drive types on the dimensions of trust. We also visualized and contrasted the trends of the five dimensions of trust across the two mobilities using (Figures 5, 6).

Survey results of five dimensions of trust are illustrated in Figure 5 (defensive) and Figure 6 (aggressive). The plots show the evolution of the average response value for each trust dimension as participants interacted with the drives in the session, starting with the baseline. Specifically, we compare ${\text{Base}}_{\text{C}}^{\text{Ses}1}$, ${\text{Tut}}_{\text{C}}^{\text{Ses}1}$, ${\text{Std}}_{\text{C}}^{\text{Ses}1}$, and ${\text{Pro}}_{\text{C}}^{\text{Ses}1}$ to ${\text{Base}}_{\text{SM}}^{\text{Ses}1}$, ${\text{Tut}}_{\text{SM}}^{\text{Ses}1}$, ${\text{Std}}_{\text{SM}}^{\text{Ses}1}$, and ${\text{Pro}}_{\text{SM}}^{\text{Ses}1}$, respectively (refer to Figure 3). For participants who experienced defensive AD behaviors, the results of the linear mixed effects model showed there was a significant effect of drive type on all five dimensions of trust. The model outputs for the fixed effect of drive type are as follows.

1. Reliability/Competence *F*_(3, 66)_ = 12.39, *p* < 0.00001, effect size $\eta_{p}^{2}=0.36$
2. Understanding/Predictability *F*_(3, 66)_ = 14.36, *p* < 0.00001, effect size $\eta_{p}^{2}=0.40$
3. Intention of Developers *F*_(3, 66)_ = 8.33, *p* < 0.0001, effect size $\eta_{p}^{2}=0.27$
4. Propensity to Trust *F*_(3, 66)_ = 10.80, *p* < 0.00001, effect size $\eta_{p}^{2}=0.33$
5. Trust in Automation *F*_(3, 66)_ = 8.18, *p* = 0.0001, effect size $\eta_{p}^{2}=0.27$.

Additionally, there is not a significant effect of mobility mode (vehicle type) on any of the dimensions of trust at the 95% confidence interval. There is no significant effect from the interaction between drive type and mobility mode on any of the trust dimensions.

**Figure 7:**
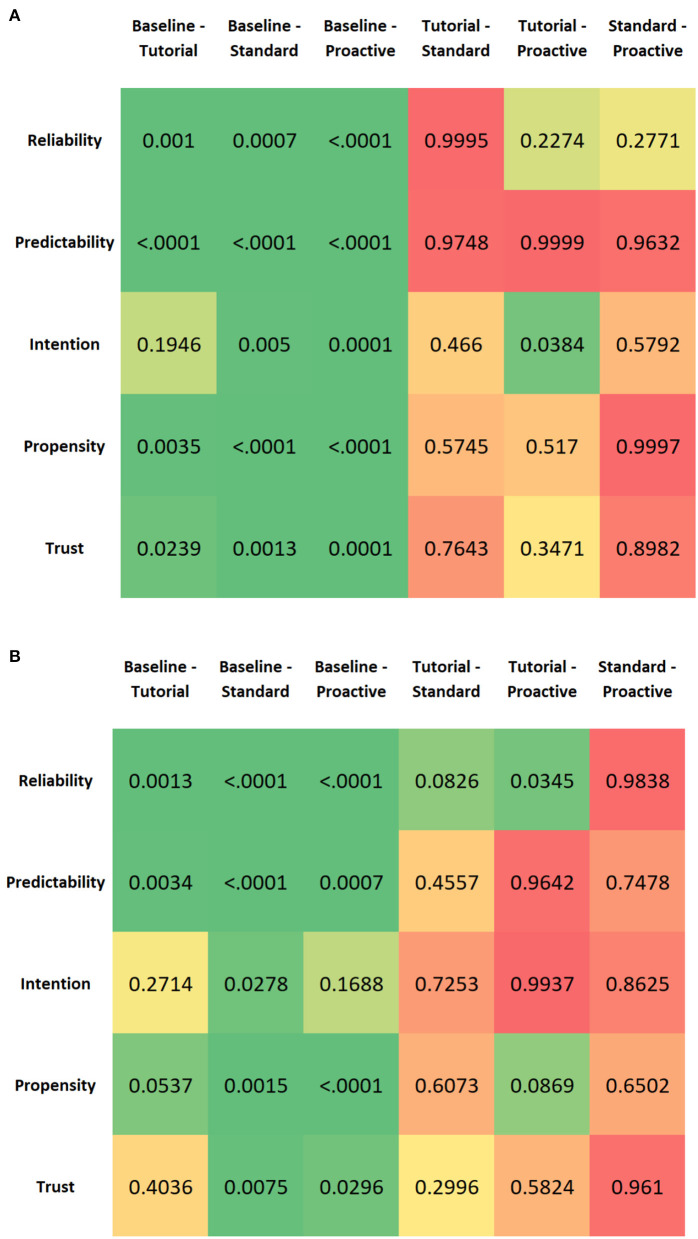
Tukey pairwise *post-hoc* tests for the drive type levels. Green indicates statistical significant comparisons whereas other colors represent non-significant comparisons. **(A)** Defensive—Session 1. **(B)** Aggressive—Session 1.

A pairwise comparison of the drive type factor was conducted using Tukey's test to understand when a significant change was measured for each dimension of trust, and the results are shown in Figure 7A. We find that the only significant pairwise comparisons were “baseline (after briefing) and tutorial,” “baseline and standard (w/o conflict events),” and “baseline and proactive (w/ conflict events)” for all dimensions of trust. This indicates that the increase in trust from the initial explanation of the AD vehicle's reliability to after the tutorial (during which the participant uses the simulator for a few minutes) is significant while trust rarely changes significantly between the tutorial, standard (w/o conflict events), and proactive (w/ conflict events) drives.

Now, if we compare the evolution of the dimensions of trust in Figure 5, all dimensions of trust, except for *propensity to trust* (Figure 5D), are higher for the automated car than the sidewalk mobility. Additionally, we see that the trust progression from baseline (when the participants were briefed about the reliability) to proactive driving at conflict events is relatively the same for both mobility modes for all dimensions of trust. This is also confirmed statistically based on the observation that no significant effect of mobility mode on any of the dimensions of trust was found. From Figure 5D, the *propensity to trust* is approximately the same for the two mobilities across all drives; this may signify that participants' *propensity to trust* a new technology for the first time does not differ between mobility modes.

For participants who experienced aggressive AD behaviors, we found that there is a significant effect of drive type on all five dimensions of trust. The model outputs for the fixed effect of drive type are as follows.

1. Reliability/Competence *F*_(3, 66)_ = 18.92, *p* < 0.00001, effect size $\eta_{p}^{2}=0.46$
2. Understanding/Predictability *F*_(3, 66)_ = 9.77, *p* < 0.0001, effect size $\eta_{p}^{2}=0.31$
3. Intention of Developers *F*_(3, 66)_ = 2.94, *p* = 0.0394, effect size $\eta_{p}^{2}=0.12$
4. Propensity to Trust *F*_(3, 66)_ = 9.21, *p* < 0.0001, effect size $\eta_{p}^{2}=0.30$
5. Trust in Automation *F*_(3, 66)_ = 4.44, *p* = 0.0067, effect size $\eta_{p}^{2}=0.17$.

For aggressive drivers, there is neither a significant main effect of mobility mode nor a significant interaction effect between drive type and mobility mode on any of the dimensions of trust. A pairwise comparison of the drive type factor is conducted using Tukey's test to understand when a significant change was measured for each dimension of trust. According to Figure 7B, for the first dimension of trust, *reliability/competence*, there is a significant increase from the baseline—the initial explanation of the autonomous vehicle's reliability—to after the tutorial. Additionally, there is a significant increase from after the tutorial to after the proactive drive, implying that the trust dimension of perceived *reliability/competence* builds as participants experience standard events followed by conflict events. *Therefore, aggressive proactive behavior in navigating conflict events promotes a reliable view of the automated driving system*.

Only one other dimension of trust, *understanding/predictability*, has a significant change in trust between the baseline and after the tutorial. This signifies that this dimension of trust does not change to the same extent during the standard (interacting with standard events) and proactive drives (interacting with conflict events). *Intention of developers* has a significant increase in trust from the baseline to after the standard drive; however, trust in the developers' intentions actually decreases slightly after the proactive drive. This shows that participants who prefer to be driven aggressively actually don't have complete confidence in the developers, likely attributable to a few aggressive actions at certain conflict events and this being the first interaction with the mobility. Lastly, *trust in automation* has a significant change in trust from baseline to standard and baseline to proactive, signifying that the participants' trust increases at first but then plateaus after the standard drive (i.e., doesn't change from the proactive drive).

### 3.2. Effect of prior experience with an automated car mobility on trust in a novel automated mobility

Thirteen of twenty-four participants who experienced the car mobility during their first session said that their trust transferred to the sidewalk mobility during their second session. Four of those twenty-four participants said that their trust did not transfer to the sidewalk mobility from the car. Seven participants either did not comment on whether their trust transferred or not or their semi-structured interview audio was not audible.

Of the thirteen participants whose trust did transfer from the car mobility to the sidewalk mobility, several shared their experience and why they felt that their trust transferred. As Participant 6 explained, “*I think after going in the car, I got to trust it and I got what I was doing, so I feel like with a scooter I expected kind of the same thing, like how it was gonna stop and speed up and how it was gonna react to people and other cars.”* Two participants reported they had positive expectations of the second mobility because they assumed both mobilities were created by the same developers. Participant 30 stated, “*If I did the car, and it seems like it was trustworthy, the same people are developing this one. So it must have the same principles.”* Some participants suggested that they had a general idea of how the second mobility would behave from a technological perspective. Participant 1 had a very good experience with the car mobility, so they said referring to the sidewalk mobility, “*if it's by the same developers and kind of works the same way, then I really have a higher level of trust and confidence in it.”*

On the contrary, four participants had a different view that trust did not transfer across mobilities. Participant 11 responded, “*I think it was pretty independent”* when asked if trust in the car mobility from session 1 influenced their trust in the sidewalk mobility in session 2. However, Participant 11 did confirm that overall they felt safer in the car. Participant 36 reiterated the sentiment shared by Participant 11, “*I don't think it (the car mobility) influenced at all like how I viewed [the sidewalk mobility]; it felt like two completely different tasks.”* Participant 34 saw the two experiences as separate due to the physical size of the mobilities and their potential of hurting someone. Lastly, Participant 32 felt like the experience in session 1 with the car mobility is in a more structured environment whereas on the sidewalk it is “*too spontaneous”* due to pedestrian variability; therefore, it felt like the two experiences were not interdependent.

To analyze the effect of the interaction with an automated car on trust in the automated sidewalk mobility, we compare the sidewalk mobility responses for the participants who interacted with the sidewalk mobility in session 2 to that of the participants who interacted with the sidewalk mobility in session 1. This allows us to evaluate whether (or not) the prior experience with an automated car affected the evolution of trust in the sidewalk mobility. To mitigate the effect of drive type, we only consider the survey results of the 6 participants who had the same standard-proactive drives on the sidewalk mobility during session 2 for comparison to that of the 12 participants with the sidewalk mobility in session 1. To investigate the statistical significance between the responses of the two groups of participants for a given drive type, we perform a two-sample *t*-test for each of the drive types for all dimensions of trust. For example, we compare ${\text{Base}}_{\text{SM}}^{\text{Ses}1}$ vs. ${\text{Base}}_{\text{SM}}^{\text{Ses}2}$, ${\text{Tut}}_{\text{SM}}^{\text{Ses}1}$ vs. ${\text{Tut}}_{\text{SM}}^{\text{Ses}2}$, ${\text{Std}}_{\text{SM}}^{\text{Ses}1}$ vs. ${\text{Std}}_{\text{SM}}^{\text{Ses}2}$, and ${\text{Pro}}_{\text{SM}}^{\text{Ses}1}$ vs. ${\text{Pro}}_{\text{SM}}^{\text{Ses}2}$ for the dimension *intention of developers*.

**Figure 8:**
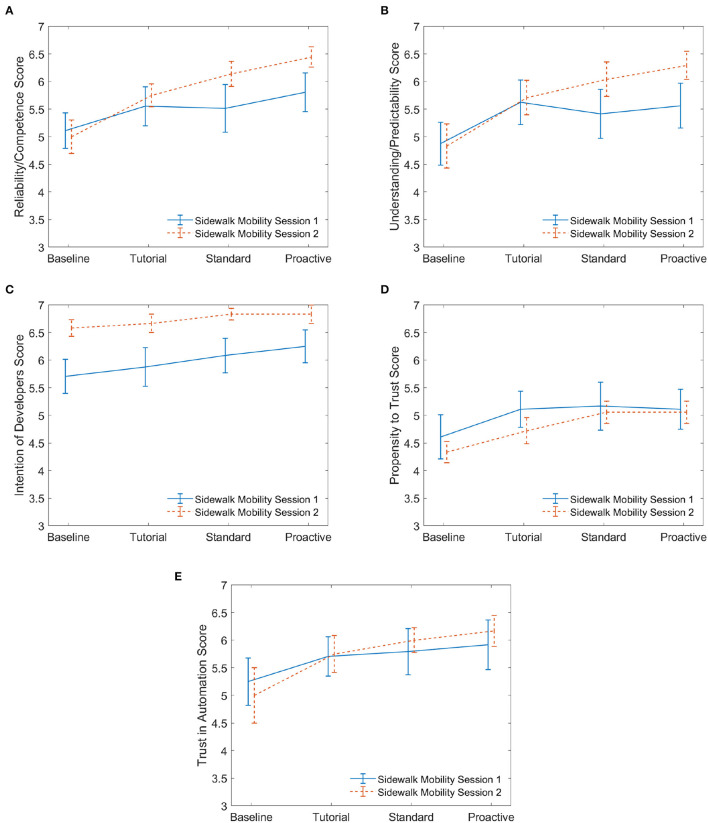
Sidewalk mobility defensive: session 1 vs. session 2. **(A)** Reliability. **(B)** Predictability. **(C)** Intention. **(D)** Propensity. **(E)** Trust.

**Figure 9:**
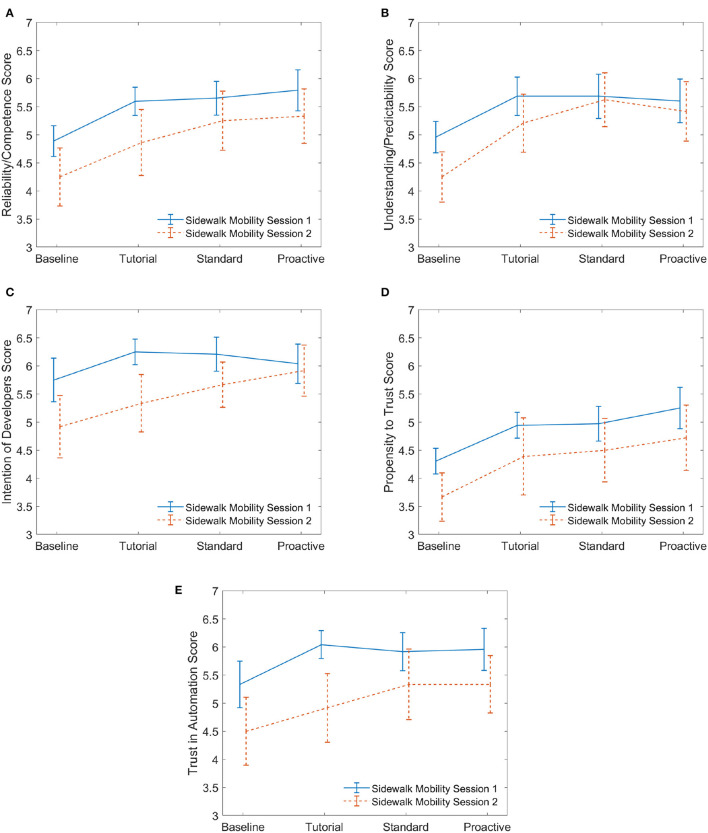
Sidewalk mobility aggressive: session 1 vs. session 2. **(A)** Reliability. **(B)** Predictability. **(C)** Intention. **(D)** Propensity. **(E)** Trust.

Figures 8, 9 show the changes in the dimensions of trust across the drives for defensive and aggressive participants, respectively. For defensive drivers (Figure 8), the two-sample *t*-test results were significant for *intention of developers*. These results are shown in Table 6. This result demonstrates that prior experience with an automated car leads to a significant trust increase with respect to the *intention of the developers* who made the automation, but no significant difference in *trust in automation* of the actual system. Figure 9 illustrates results for participants who experienced aggressive AD behaviors. None of their two-sample *t*-test results were significant, implying that aggressive driving style of cars did not influence the participants' trust in the sidewalk mobility.

**Table 6:** Two sample *t*-test results for *intention of developers* for comparing sidewalk mobility session 1 to sidewalk mobility session 2; all other dimensions of trust did not have any significant differences.

	**Session 1 mean**	**Session 2 mean**	**t-statistic**	**df**	***p*-value**
Base	5.708	6.583	−2.525	15.06	0.0233
Tutorial	5.875	6.667	−2.048	14.89	0.0587
Standard	6.083	6.833	−2.272	13.26	0.0403
Proactive	6.250	6.833	−1.707	15.60	0.1077

### 3.3. Observations and thoughts on transparency

During the semi-structured interview, many participants discussed trust in different automated mobilities. Apart from trust, one emergent theme from the interviews was the importance of transparency. For this study, bounding boxes highlighted other vehicles and pedestrians on the same side of the road. Additionally, vehicles provided voice alerts during proactive events. 14/48 participants expressed positive opinions on voice alerts, as it helped them understand the mobility's current and future actions. One participant stated, “*[the voice assistance] is double assurance that the system is actually. . .keeping up with everything; and it's not going to run over someone.”* However, some participants expressed concerns about the promptness of the voice assistance, and reported that it came too late in order to be helpful. 10/48 participants found the voice alerts barely perceptible, easily ignored, or redundant. A participant stated “*Maybe at the beginning [it was helpful], like it helped me know what was going to happen. But at the end. . .I knew what was going to happen, so it was very unnecessary.”* These observations highlight the importance of automation using variable or adaptive transparency. When users experience automation initially, greater amounts of transparency are necessary to help increase their trust; however, as expressed by some participants, a continuously high level of transparency can become redundant or distracting.

In summary, the first research question explored how human trust evolved in one automated mobility vs. another automated mobility with no prior interaction with either of the mobilities. Findings suggest that while trust increases over time, the effect of mobility was not significant. The second research question explored how prior experience in an automated car influence trust in a novel automated mobility (that is not a car). Findings suggest that only the intention of developers was found to be significant.

## 4. Discussion

### 4.1. Key findings

We found that during an initial session with either a car or sidewalk mobility, all five dimensions of trust increased similarly. These dimensions are reliability, predictability, intention of developers, propensity to trust, and trust in automation. This suggests that there may not be differences in how trust builds in an automated car vs. an automated sidewalk mobility. Additionally, we observed that participants who preferred a defensive automated driving style had a significantly higher amount of trust in the *intention of developers* while using the sidewalk mobility during their second session vs. their defensive counterparts using the sidewalk mobility during their first session. Overall, participants confirmed in their semi-structured interviews that their trust increased from the first mobility to the second.

Another important finding from this work is that *vehicle* type had no effect on any dimensions of trust as shown in Section 3.1.3. This result suggests that users' trust can grow and evolve across different mobilities even when they are unfamiliar with a particular mobility. This has important implications for several stakeholders in a future MaaS ecosystem, in which it is expected that users will frequently interact or transition between different mobility types. As mentioned in Section 3, some participants reported that their trust transferred from the first mobility to the second due to an assumption that the mobilities were made by the same “company”, or “developers”, or use the same “software”, “system”, or “artificial intelligence”. This suggests that brand loyalty to automakers, which certainly influences consumers as they make personal vehicle purchasing decisions (Jørgensen et al., 2016; Nadzri et al., 2016), may also extend to users' trust in automated mobilities, even when the “vehicle” is not a conventional car or other types of automobile.

Interestingly, we also found that certain *drive* types had a significant effect on all dimensions of trust. Specifically, there was a statistically significant effect on all dimensions of trust for participants when we compared their baseline reported trust to that after they experienced the standard drive. Drives with defensive actions had the most consistent and significant effects on trust. Drives with aggressive actions decreased trust in the intention of the developers, likely due to what could be perceived as dangerous actions at certain events. These results suggest that trust can increase or decrease in accordance to the type of experience a user has in a given drive. Similar to purchasing a new car, users may need opportunities to “test drive” and positively experience new forms of mobility. Education campaigns have already been developed for e-bikes and e-scooters in cities including New York City in response to increasing safety concerns (Hu and Marcius, 2021). Automated mobilities could potentially exacerbate safety concerns when sharing sidewalks and roadways with electric or manually-powered personal mobility devices. Therefore, considering a future MaaS ecosystem, cities in particular may need to consider training or education programs for residents on varied mobility types, both to improve safety and adoption.

The trends shown in Figure 6 (aggressive) indicate that the baseline levels of trust in the sidewalk mobility are similar to the baseline in Figure 5 (Defensive). What differs are the trends seen in the car mobility. With the car being the more familiar form of mobility, the human's trust dynamics when experiencing aggressive actions exhibit similar trends to an unfamiliar form of mobility—the sidewalk mobility. This finding indicates that the aggressive driving experience in a car can be likened to that of something unfamiliar and thereby influence trust. Interestingly, although not significant, we see trends from Figure 6 that imply individuals who preferred aggressive driving showed an increased willingness to rely on and trust the car despite the fact that their trust in the car's predictability decreased when they encountered sequential proactive behavior. This suggests that proactive automated driving can increase trust and reliance in a car mobility which implies “faith,” or a higher level of trust, in the automated car. The introduction of “conflict” events in proactive drives was a novel aspect of the experiment as it served to stimulate changes in trust in ways that were more consistent with real driving scenarios. Therefore, this finding is especially encouraging in that it shows the participants may trust more advanced decision-making on the part of an automated vehicle.

Lastly, the use of voice prompts to increase automation transparency was helpful and reassuring to some participants. However, participants reported that while the transparency was appreciated initially, it became a distraction as participants became more familiar with each mobility. This provides a strong motivation for the use of adaptive transparency (Akash et al., 2020b) that is sensitive both to individual user's preferences as well as time.

### 4.2. Limitations of the study

It is likewise important to highlight some limitations of the work presented here. First, certain aspects of the vehicle simulators could be changed to improve ecological validity. To mitigate the negative effects of simulator glitches, each drive was pre-recorded. While this ensured a consistent experience for each participant, it did not allow participants to actually takeover control of the vehicle. Instead, we were limited to only measuring “intent to takeover”. The second aspect that should be revisited is the smoothness of each mobility. The car simulator intentionally had a smoother ride than the sidewalk mobility for realism. However, some participants reported that the dynamics of the sidewalk mobility were too bumpy during some portions of the drives. This could have introduced confounds in the users' experience. Therefore, the settings on each simulator should be adjusted in future experiments. For observing individual differences and demographic factors, future studies should consider controlling for differences in age, gender, and culture to understand if those factors influence trust across mobilities. While countermeasures in our experimental design did not introduce bias due to participant fatigue and affect, future studies could account for fatigue and affect for measuring trust in automation. Finally, a limitation of this study is the small sample size of participants who experienced the sidewalk mobility in session 2 that we could compare with those who experienced it in session 1. To validate the differences observed in this comparison, follow-up experiments with a larger sample size should be conducted.

Nevertheless, despite these limitations, this dual mobility experiment and associated analysis represents the first investigation of trust transfer across different automated mobility types and serves as a foundation for future work aimed at enabling a shared mobility future.

## 5. Conclusion

Motivated by a rapidly changing transportation ecosystem in which it is expected that users will transition between different automated mobility types, we conducted a novel dual mobility experiment to explore how trust evolves and transfers across an automated car and an automated sidewalk mobility. We used both quantitative and qualitative methods to measure trust, including trust questionnaires throughout the experiment and a semi-structured interview at the conclusion of the experiment. Our analyses yielded new insights into the effect of mobility and prior interactions on trust among mobility users.

A major contribution of this work is the development of a novel dual mobility experiment that provided a means for studying trust in and across multiple mobilities. The mobility platforms combined with the motion base allowed us to compare how trust evolved in an automated sidewalk mobility vs. in an automated car mobility. Additionally, we investigated how trust transferred from a car mobility to a sidewalk mobility. The design and inclusion of novel conflict events in our simulator study permitted a deeper investigation of trust, seeing as these events did not depend upon varying the automation's reliability. To date, very few studies have included conflict events with micromobility as ego vehicles in their simulator experiments (Bella and Silvestri, 2018; Deliali et al., 2021). The empirical results of our study provide new insights into user experience in automated technologies, particularly as it relates to trust. The findings are particularly useful for human-computer interaction researchers, MaaS developers, and those working on highly automated mobilities.

## Data availability statement

The raw data supporting the conclusions of this article will be made available by the authors, without undue reservation.

## Ethics statement

The studies involving human participants were reviewed and approved by Institutional Review Board at San Jose State University. The patients/participants provided their written informed consent to participate in this study. Written informed consent was obtained from the individual(s) for the publication of any identifiable images or data included in this article.

## Author contributions

KA, TM, NJ, TR, AK, MK, JH, and NM: study conception and design. KA, ZZ, AK, EU, AMo, JS, and AMe: data collection. AK, EU, AMe, SM, KA, NJ, MK, JH, TM, and ZZ: analysis and interpretation of results. SM, KA, NJ, JH, and TR: draft manuscript preparation. All authors contributed to the article and approved the submitted version.
